# Breast and cervical cancer in transgender men: literature review and a case report

**DOI:** 10.1177/17588359241259466

**Published:** 2024-08-10

**Authors:** Francesca Sofia Di Lisa, Alice Villa, Lorena Filomeno, Teresa Arcuri, Benito Chiofalo, Giuseppe Sanguineti, Laura Pizzuti, Eriseld Krasniqi, Maddalena Barba, Domenico Sergi, Francesco Lombardo, Francesco Romanelli, Claudio Botti, Giovanni Zoccali, Gennaro Ciliberto, Patrizia Vici

**Affiliations:** Phase IV Clinical Studies Unit, IRCCS Regina Elena National Cancer Institute, Rome, Italy; Department of Experimental Medicine, Sapienza University of Rome, Rome, Italy; Phase IV Clinical Studies Unit, IRCCS Regina Elena National Cancer Institute, Via Elio Chianesi 53, Rome 00144, Italy; Phase IV Clinical Studies Unit, IRCCS Regina Elena National Cancer Institute, Rome, Italy; Medical Oncology A, Policlinico Umberto I, Department of Radiological, Oncological and Anatomo-Pathological Sciences, Sapienza University of Rome, Rome, Italy; Gynecologic Oncology Unit, Department of Experimental Clinical Oncology, IRCCS Regina Elena National Cancer Institute, Rome, Italy; Department of Radiation Oncology, IRCCS Regina Elena National Cancer Institute, Rome, Italy; Division of Medical Oncology 1, IRCCS Regina Elena National Cancer Institute, Rome, Italy; Phase IV Clinical Studies Unit, IRCCS Regina Elena National Cancer Institute, Rome, Italy; Division of Medical Oncology 1, IRCCS Regina Elena National Cancer Institute, Rome, Italy; Division of Medical Oncology 2, IRCCS Regina Elena National Cancer Institute, Rome, Italy; Department of Experimental Medicine, Sapienza University of Rome, Rome, Italy; Department of Experimental Medicine, Sapienza University of Rome, Rome, Italy; Division of Breast Surgery, IRCCS Regina Elena National Cancer Institute, Rome, Italy; Department of Plastic and Reconstructive Surgery, IRCCS Regina Elena National Cancer Institute, Rome, Italy; Scientific Direction, IRCCS Regina Elena National Cancer Institute, Rome, Italy; Phase IV Clinical Studies Unit, IRCCS Regina Elena National Cancer Institute, Rome, Italy

**Keywords:** case report, breast and cervical cancer, screening, testosterone therapy, transgender men

## Abstract

Transgender individuals exhibit a higher prevalence of cancer-related risk factors, such as substance abuse and sexually transmitted infections. These factors, coupled with suboptimal adherence to cancer screening recommendations, may lead to a higher incidence of cancers, such as breast and cervical cancer, and contribute to delayed diagnoses in transgender patients. Herein, we report a unique case of a transgender man with a history of alcohol and drug abuse, undergoing gender-affirming exogenous testosterone therapy, who developed synchronous locally advanced breast cancer and human papilloma virus (HPV)-related cervical cancer. He underwent concurrent chemoradiation for cervical cancer and surgery followed by endocrine therapy for breast cancer. The treatments were suboptimals due to patient’s comorbidities, among them liver cirrhosis leading to an early death. Additionally, we have conducted a review of existing literature, including case reports, clinical studies, and review articles investigating the role of potential risk factors specifically related to breast and cervical tumors in transgender men. Gender-affirming testosterone therapy is common among transgender men to induce gender affirmation, but its link to breast cancer risk remains ambiguous, with studies being limited and sometimes contradictory. Conversely, HPV is a well-established cause of up to 99% of cervical cancers. Despite persistent risk for cervical cancer in transgender men who retain their cervix, several studies indicate notable disparities in screening adherence, due to personal and structural barriers. Moreover, alcohol and drug use disorders, commonly encountered in transgender population, may negatively influence the adherence to screening programs. Current cancer screening guidelines for this population are somewhat unclear, and specific programs based on more robust data are urgently required along with further tailored studies.

## Plain language summary

Breast and cervical cancer in transgender men: literature review and a case report

Transgender individuals are persons whose gender identity does not conform to that typically associated with the sex to which they were assigned at birth. Transgender people may have more cancer-related risk factors, such as substance abuse and sexually transmitted infections. These factors, along with suboptimal adherence to cancer screening, may lead to a higher incidence of cancers, among them breast and cervical cancer, and may also contribute to delayed diagnoses. Herein we report the case of a transgender man, recorded as female at birth but identifying as male, with a history of alcohol and drug abuse. He underwent testosterone therapy in order to affirm his gender. Moreover, he refused cancer screening, due to personal and social barriers. During the transition, he developed simultaneously a locally advanced breast cancer and a cervical cancer, the latter related to an infection from Human Papillomavirus. The patient was treated with chemoradiation for cervical cancer, and with surgery followed by endocrine therapy for breast cancer. The treatments were suboptimals due to patient’s comorbidities, partly related to his previous lifestyle. Additionally, we have conducted a review of existing literature on the topic. Trangender men usually undergo testosterone to induce gender-affirmation. The role of testosterone therapy in breast cancer development remains unclear, with studies being contradictory. Conversely, Human Papillomavirus is a well-established cause of cervical cancers. Transgender men who retain their cervix are at risk for cervical cancer. Despite the persistent risk, notable disparities in screening adherence, due to personal and structural barriers, are reported. Moreover, alcohol and drug use disorders, commonly encountered in transgender population, may contrtibute to the low adherence to screening programs. Furthermore, screening guidelines are somewhat unclear, and specific programs are urgently required.

## Introduction

Gender dysphoria describes a distress experienced by a person related to a mismatch between gender identity and the sex assigned at birth.^1^ People with gender dysphoria commonly identify as transgender. This type of distress may lead to anxiety, depression, alcohol and drug abuse, eating disorders, and promiscuous sexual behavior,^2^ some of which are risk factors for several types of cancer. In particular, substance use and abuse, smoking, and sexually transmitted infections, along with a suboptimal adherence to cancer screening recommendations, may contribute to delayed diagnosis and the development of specific types of cancer.^3,4^ People with gender dysphoria frequently undergo gender-affirming treatments, including hormone therapy, surgery, and mental healthcare.^5^ Moreover, the role of exposure to high-dose and prolonged hormone therapy remains of topic of intense debate.^4^

Breast cancer is the most common malignant tumor in cisgender women and the second leading cause of cancer mortality.^6^ However, breast cancer risk in specific subpopulations, such as transgender people, is not yet fully characterized. Some evidence suggests an increased breast cancer risk in transgender men compared to their cisgender counterpart, while having a lower risk than cisgender women.^7^ Transgender assigned female at birth (AFAB) individuals, recorded as female at birth but identifying as male, may undergo gender-affirming androgen therapy to align their physical characteristics with their gender identity. Testosterone is commonly used to induce and maintain masculinization. However, the effects of androgens on the mammary gland tissue remain unclear and largely unexplored. Only a few reports are available on breast cancer in transgender patients previously and/or currently exposed to testosterone, with some suggesting a potential but inconclusive association between higher doses of testosterone and increased breast cancer risk.^8^

High-risk human papilloma viruses (HPVs) are sexually transmitted and cause cervical cancer. Transgender people may experience sexual promiscuity, a behavior associated with an increased risk of sexually transmitted infections, including HPV-related diseases, human immunodeficiency virus, and others.^9,10^ Moreover, whether testosterone plays a role in HPV infection and/or cervical cancer development remains largely unexplored.^11^

Synchronous primary breast and cervical cancer are uncommon at the general population level.^12^ Herein, we report on the first case, to our knowledge, of a transgender man who developed breast and cervical cancer simultaneously, with both cancers presumably related to a lack of screening and increased risk factors. The reporting of this patient’s clinical case conforms to the CARE case report guideline and is provided as a Supplemental Material 1.^13^

## Case description

We report the case of a 61-year-old transgender man who developed two typically female cancers simultaneously: invasive breast cancer and locally advanced cervical cancer.

The patient denied any family history of cancer, except for prostate cancer. The germline BReast CAncer gene 1 and 2 (BRCA1/2) status was unknown, due to the patient’s refusal to undergo testing. Concerning the gynecological history, the patient referred nulliparity and menopause onset at the age of 50 years. He denied smoking behavior. Past medical history included several years of drugs and alcohol abuse, followed by a diagnosis of hepatitis C in 1995, which was treated with an 18-month course of sofosbuvir several years later.

From May 2021 through April 2022, the patient underwent testosterone treatment (testosterone gel, four pump actuations, once daily) to induce masculinization. In March 2022, due to vaginal bleeding, a pelvic ultrasound and a magnetic resonance imaging were performed, showing a cervical mass of more than 3 cm, with right parametrium involvement, and extension to the vagina. In April 2022, a cervical biopsy confirmed the diagnosis of locally advanced grade 3 squamous cervical cancer, HPV+ and p16+.

**Figure 1.**
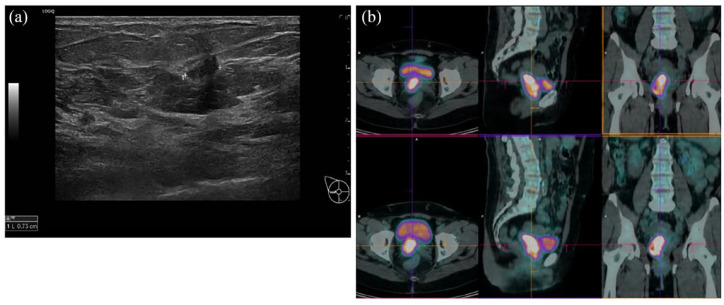
(a) Breast ultrasound: heterogeneous background echotexture with a hypoechoic area of 7 mm diameter. (b) PET-CT with FDG, performed for the initial cancer staging on April 2022, identified a hypermetabolic pathological tissue of 35 × 58 cm^2^ on the right side of the cervix, with extension to the vagina and the right parametrium. FDG, fluorodeoxyglucose; PET-CT, positron emission tomography/computed tomography.

Simultaneously, a palpable right-sided breast mass of 3 cm, located in the upper outer quadrant, was discovered through breast ultrasound [Figure 1(a)] and mammography, revealing a heterogeneous background echotexture with a hypoechoic area of 7 mm diameter in the breast without axillary node involvement. The ultrasound-guided breast biopsy revealed an infiltrating ductal carcinoma of grade 2 in the right breast, with estrogen receptor (ER) 90%, progesterone receptor (PgR) 90%, human epidermal growth factor receptor 2 (HER2) 2+, *in situ* hybridization not amplified, and a Ki 67 index of 28%.

A positron emission tomography/computed tomography (PET-CT) with fluorodeoxyglucose found no distant metastases, confirming the presence of pathological cervical neoplastic tissue with high metabolic activity, extending to the vagina [Figure 1(b)]. No focal areas of hypermetabolism were detected in the breast.

Following multidisciplinary team consultation, the decision was made to treat the cervical cancer first and to postpone breast cancer treatment.

In May to June 2022, the patient underwent concurrent chemoradiation. This included weekly intravenous administrations of cisplatin at a dose of 50 mg/m^2^, accompanied by radiotherapy. The radiotherapy was delivered using the volumetric modulated arc therapy (VMAT) technique, targeting the pelvic and inguinal nodes. The initial radiotherapy dosage was 45 Gray (Gy) in 25 fractions, followed by a boost, bringing the total radiation dose to 55 Gy. The patient developed significant hematological toxicity, with persistent thrombocytopenia (possibly related to pre-existent liver dysfunction), which led to platinum discontinuation after the second week of treatment. In July 2022, concurrent chemoradiation was followed, by intracavitary brachytherapy. During radiation therapy, the patient also developed severe diarrhea, which only partially resolved after several months, as well as mild leukopenia and persistent thrombocytopenia. A PET-CT, carried out in September 2022, showed a reduction of the cervical mass but persistent parametrial involvement.

Subsequently, in September 2022, the patient underwent bilateral nipple sparing mastectomy and right axillary dissection, with surgical pathology confirming the diagnosis of grade 3 right-sided breast infiltrating ductal carcinoma, measuring 4 cm in diameter with lymphovascular invasion. Two out of 18 axillary nodes were metastastic with extracapsular extension (pT2pN1a, G3). The immunohistochemical profile confirmed a high expression of ER and PgR, HER2 negative status, 10% expression of the androgen receptor (AR), and a 25% Ki-67 rate.

In November 2022, a PET-CT showed a substantial stability of the cervical lesion and the absence of distant metastasis from both cancers. Due to the poor tolerability of previous chemotherapy for cervical cancer, breast adjuvant chemotherapy was excluded from the treatment plan, opting instead for only the adjuvant aromatase inhibitor anastrozole. Initially, there was a plan to administer the cyclin-dependent kinase 4/6 inhibitor (CDK4/6i) abemaciclib, which was ultimately not started due to persistent diarrhea and thrombocytopenia.

From the patient’s perspective, he expressed profound disappointment upon being diagnosed with cancers affecting the breast and the cervix, two organs he had long rejected and denied due to gender dysphoria. In fact, he had previously refused to adhere to any cancer screening programs, dismissing the risk of cancer development. The patient had experienced gender dysphoria since childhood but only decided to pursue gender-affirming therapies only several decades later. Throughout his life, he endured severe psychological distress, together with alcohol and drug abuse, and sexual promiscuity. However, after the deciding on gender reassignment, he started psychotherapy together with hormonal treatments. Following the cancer diagnoses, he expressed fear about halting testosterone therapy and its potential impact on his body. Nevertheless, he agreed to temporarily interrupt testosterone administration and to accept a cancer treatment plan specifically tailored to his comorbidities and preferences.

In February 2023, due to the rapid onset of abdominal distension, the patient underwent an ultrasound and a subsequent CT scan, which revealed ascites, compensatory collateral circulation, including esophageal varices, a markedly cirrhotic liver, and sloping edemas. Based on the imaging findings and reported symptoms, a diagnosis of severe liver failure was made and the patient was referred to a hepatology center.

In May 2023, a PET-CT demonstrated multiple suspicious lung micronodules and an increase in the extent of cervical cancer infiltration into the right parametrium. A chest CT scan with contrast medium followed by a biopsy was recommended to investigate the nature of the lung micronodules.

**Figure 2.**
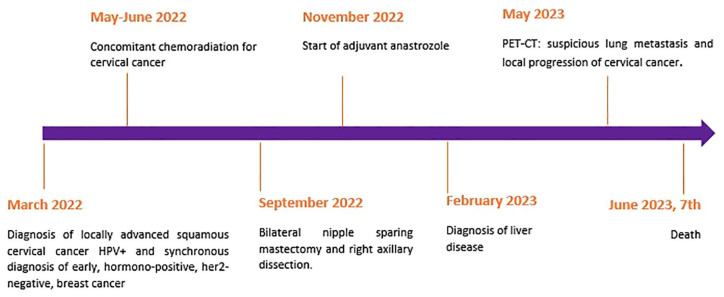
Patient medical history timeline.

Unfortunately, the patient died quickly at home, due to bleeding from esophageal varices. The timeline of cancer history is graphically displayed in Figure 2.

## Discussion

Transgender men are recorded as female at birth but identify as males and may undergo gender-affirming procedures and therapies, including surgery and/or hormone therapy. In more detail, transgender people may undergo masculinization aided by testosterone therapy, and/or choose to undergo gender-affirming surgeries (GAS), including ‘topsurgery’ (subcutaneous mastectomy or chest contouring), ‘bottomsurgery’ (phalloplasty, metoidioplasty, hysterectomy, and oophorectomy).^14,15^ While the association between top surgery and decrease in breast cancer risk is well known, the relationship between testosterone therapy and risk of cancer remains unclear. It is noteworthy that there is a lack of adequately sized and specifically tailored prospective studies of transgender population and risk of cancer, with the majority of data coming from case reports.^4,16^ The heterogeneity among transgender people and across the available studies limits the ability to provide reliable estimates of cancer risk in the transgender population.^4,17^

There are some recommendations for transgender people concerning breast cancer screening. In transgender men who have not undergone top surgery the UCSF Center of Excellence for Transgender Health, and the Guidelines from the Endocrine Society Clinical Practice and American Cancer Society recommend following the same screening protocols as those for cisgender women.^18,19^ The American College of Radiology published the Appropriateness Criteria for transgender breast cancer screening, evidence-based guidelines for specific conditions, such as transgender men with top surgery or no chest surgery, and considering age, family history of breast cancer, precancerous lesions, genetic predisposition.^20^ Overall, the guidelines recommend substantially the same screening procedures as those for cisgender women, but usually trans people, and in particular transgender men, do not adhere to them, because of gender dysphoria, feeling of discrimination against their masculine appearance, and sometimes, because of fear of being discriminated by the medical staff.^21^ The lack of reliable cancer risk estimates, along with the somewhat confusing screening programs dedicated to transgender, and the lack of adherence, results in frequent delays in cancer diagnosis.^22–25^

Breast cancer onset in transgender people may be related to multiple factors. One of the most discussed topics is the impact of exogenous testosterone on breast cancer risk. The relationship between testosterone administration, as well as endogenous testosterone secretion, and breast cancer risk remains controversial.

In women, androgens are produced in small amounts by the ovaries and adrenal glands. In postmenopausal women, ovarian and adrenal gland progressively reduce androgens synthesis, with deleterious effect on quality of life with sexual desire disorder, arousal, anorgasmy, and dyspareunia.^26^ Several studies demonstrated the utility of testosterone administration in improving total satisfying sexual activity and libido, alone or in combination with estrogen and/or progesterone.^27–29^ Conversely, the use of testosterone in premenopausal women is not advisable, due to lack of supporting evidence.^30^ Moreover, long-term safety data are lacking.^26,31^ In reference to breast cancer, it is worth mentioning, for the long-term follow-up and the population size, a 10-year prospective cohort study, analyzing data from 1267 pre- and postmenopausal women treated with long-term subcutaneous testosterone or testosterone combined with anastrozole; the study did not show an increased incidence of invasive breast tumors.^32^ Furthermore, randomized controlled trials (RCTs) exploring the association between breast cancer incidence and exogenous testosterone are very limited and often used surrogate markers, that is, mammographic density and breast cell proliferation assessed on fine needle aspiration biopsy.^33,34^ However, no RCTs on exogenous testosterone intake include a large number of patients and have long-term follow-up to establish a clear correlation between testosterone and breast cancer occurrence.^35^ In regard to endogenous testosterone production, some prospective studies showed a possible association between circulating levels of steroid hormones, including testosterone, and breast cancer risk, both in premenopausal and postmenopausal women.^36–38^ Conversely, some *in vitro* cultures and *in vivo* studies demonstrated that testosterone has antiproliferative and pro-apoptotic effects on breast tissue, without any change in mammographic breast density,^39,40^ thus apparently reducing breast cancer risk.^41^

In reference to transgender people, it is still unclear whether the administration of exogenous steroid hormones influences breast cancer onset.^17,42,43^ A large-scale retrospective cohort study was carried out in the Netherlands including 1229 transgender men and 2260 transgender women who underwent gender-affirming hormone treatment. The aim of the study was to investigate the incidence and the characteristics of breast cancer in transgender individuals. In reference to the transgender men cohort, four (*N*: 4) cases of invasive breast cancer were diagnosed. The median age at diagnosis was 47 years (range 35–59) and breast cancer occurred after a median of 15 (range 2–17) years of hormone treatment. In transgender men with breast cancer, the median estradiol level was similar to the whole cohort of transgender men examined, while the median testosterone level was lower. The study showed a lower risk of breast cancer in transgender men compared to cisgender women and a higher risk compared to cisgender men. Because of the low absolute overall risk of breast cancer in transgender people, the authors suggest to follow screening guidelines as for cisgender people.^17^ Recently, a systematic review and meta-analysis on risk and incidence of breast cancer in transgender individuals was published.^7^ Data from 6 cohort studies and 35 case reports showed that breast cancer risk is higher in transgender people, both men and women, compared with cisgender men. Conversely, the risk appears to be lower than cisgender women. A further systematic review including 76 studies confirmed a higher breast cancer risk in transgender men compared with cisgender men, but the risk was lower compared with cisgender women. This may suggest a possible role of AR stimulation or testosterone conversion to estradiol.^44^ In addition, Silverberg *et al.*^45^ showed higher rates of breast cancer in transgender men in comparison to male referents, but the risk did not differ significantly in comparison with the matched female cohort. Table 1 summarizes the most relevant studies reported, in reference to the administration of exogenous hormones and cancer risk.

**Table 1.** Most relevant studies reported in the case report, in reference to the administration of exogenous hormones and cancer risk.

Type of study	Type of exogenous hormone	Population	Number	Objective	Conclusions	References
Systematic review and meta-analyses	GAHT (not specified)	Transgender people (women and men)	6 cohort studies plus 35 case reports	Incidence and breast cancer risk quantification	FtM and MtF individuals are at substantially higher risk of developing BC in comparison to cisgender men, though at lower risk than cisgender women. These individuals should periodically undergo breast or chest examinations	Corso *et al.*^7^
Retrospective cohort study.	GAHT (not specified)	Transgender people (women and men)	2260 trans women and 1229 trans men	To investigate the incidence and characteristics of BC in transgender people in the Netherlands compared with the general Dutch population	Increased risk of BC in trans women compared with cisgender men, and a lower risk in trans men compared with cisgender women	De Block *et al.*^17^
Double-blind, placebo-controlled RCT	Testosterone *versus* placebo	Postmenopausal cis-gender women	814 women	Evaluation of the change from baseline to week 24 in the 4-week frequency of satisfying sexual episodes	Modest but meaningful improvement in sexual function. Uncertain long-term effects of testosterone, including effects on the breast	Davis *et al.*^28^
Prospective cohort study	Subcutaneous testosterone implants or testosterone implants combined with anastrozole	Pre and postmenopausal cis-gender women	1267 women	Investigation of the incidence of invasive BC in pre and postmenopausal women (presenting with symptoms of androgen deficiency) treated with subcutaneous testosterone implants or testosterone implants combined with anastrozole	Long-term therapy with subcutaneous testosterone, or testosterone combined with anastrozole, did not increase the incidence of invasive BC	Glaser *et al.*^32^
Double-blind, placebo-controlled RCT	TTP 150 µg/d, or TTP 300 µg/d, *versus* placebo	Postmenopausal cis-gender women	279 women	To compare effects of two doses TTP with placebo in postmenopausal women without concomitant EPT on mammographic density over 52 weeks	TTP therapy appears to have no significant effect on digitally quantified absolute or percent dense mammographic area	Davis *et al.*^33^
Double-blind, placebo-controlled RCT	2 mg/norethisterone acetate 1 mg plus testosterone patch releasing 300 µg/24 h or placebo patch	Postmenopausal cis-gender women	99 women	To study the effects of testosterone addition on breast cell proliferation during postmenopausal estrogen/progestogen therapy	Addition of testosterone may counteract breast cell proliferation as induced by EPT in postmenopausal women	Hofling *et al.*^34^
RCT	TTP *versus* placebo	Postmenopausal cis-gender women	967 women	Safety and tolerability of testosterone patch therapy for up to 4 years in surgically menopausal women with hypoactive sexual desire disorder	There was no increase over time in the rate of new occurrences or severity of AEs, serious AEs, or withdrawals due to AEs. Consistent with age-appropriate expected rates, three cases of invasive breast cancer were observed	Nachtigall *et al.*^41^
Systematic review	All forms of testosterone- or estrogen-containing GAHT	Transgender people (women and men)	7 cohort studies, 2 cross-sectional studies and 34 case reports	To determine whether tumor risk in transgender individuals differs from the general population, to guide clinical screening recommendations	Retrospective cohort studies suggest no increase in risk of tumor development in transgender individuals receiving GAHT compared to the general population (mean ages of cohorts young and affirming hormone treatment for insufficient durations to assess tumor risk). Case reports raise potential associations between high-dose oestradiol and anti-androgen therapy with prolactinoma and meningioma, respectively	McFarlane *et al.*^42^
Systematic review	GAHT (not specified)	Transgender people (women and men)	43 articles	To assess breast and reproductive cancer prevalence in the transgender population. To elucidate any associations between GAHT and risk of these cancers	GAHT have not been shown to affect cancer risk, but there is a clear need for well-designed, robust studies to confirm or refute this	Joint R *et al.*^43^
Systematic review	Testosterone GAHT	Transgender men	76 studies	To investigate the impact of exogenous testosterone on BC risk in transmasculine individuals	Transgender men may have decreased risk for BCa when compared with cisgender women; however, any BC that does occur may have different clinical presentations and underlying mechanisms compared with cisgender women and men.	Gurrala *et al.*^44^

AEs, adverse events; BC, breast cancer; EPT, estrogen-progestogen therapy; FtM, female to male; GAHT, gender-affirming hormone therapy; MtF, male to female; RCT, randomized clinical trial; TTP, testosterone transdermal patch.

Also, considering the data from the literature and identified case reports, to our knowledge, only 26 cases of breast cancer in transgender women and 25 cases in transgender men have been published so far.^3^

Based on current evidence, it is not possible to conclude about the associations between transgender men using testosterone therapy and breast cancer development. Moreover, transgender individuals face numerous barriers to healthcare, such as stigmatization, discrimination, inability to pay, and other socioeconomic barriers, consequently not adhering to screening programs with delays in diagnosis.^21^

HPV is the most common sexually transmitted virus, transferred by genital, anal, oral mucosal contact.^46^ The related risk factors include the number and behavior of sexual partners, socioeconomic status, age, race, tobacco smoke, and education level.^47^ HPV infection can occur across different mucosal sites at various anatomical districts and has the potential to promote cell proliferation and malignant transformation.^48,49^ Adequate cervical cancer screening, with early detection of precancerous lesion, together with HPV vaccines and public health initiatives focused on modifiable risk factors have shown efficacy in reducing HPV-related death.^50^

Despite the persistent risk for cervical cancer in transgender men who retain their cervix and recommendations for screening, several studies indicate disparities in screening adherence in transgender men compared to cisgender women.^51^ In more detail, transgender men avoid cervical screening due to intrapersonal and structural barriers, such as stigma and discrimination, thereby increasing their risk of developing cervical cancer.^52^ Moreover, Peitzmeier *et al.*^53,54^ showed, in a retrospective chart review, that transgender men were 37% less likely to be up-to-date with their Pap test than cisgender individuals, and more likely to have an inadequate cervical sampling. To our knowledge, only one single RCT, investigating the performance and acceptability of self-collected vaginal specimens for HPV detection in transgender men, reported the prevalence of HPV infection in this population. Data showed a 16% high-risk HPV positivity, which is comparable to cisgender women.^55^

In addition to secondary prevention, primary prevention of HPV-related cancers is available with immunization against HPV through vaccination.^56^ However, the rates of HPV vaccination in the transgender male individuals are unknown.^57^

Even tobacco smoke, which is prevalent among transgender people,^58^ is a well-established risk factor for precancerous lesions and cervical cancer, contributing to carcinogenesis.^59^ Conversely, no clear evidence has been reported regarding the association between testosterone therapy and development of cervical cancer in transgender men; there are only some contradictory reports on abnormal Pap smears compared to cisgender women.^60^

Overall, to our knowledge, only three cases of cervical cancer in transgender men have been reported in the medical literature.^61–63^ Despite the rarity of these events, periodic cervical screening must be strongly recommended for transgender men who have decided to permanently retain the cervix or are doing so while waiting for GAS.

Moreover, transgender people, especially those experiencing gender discrimination, often suffer from alcohol and drugs abuse. Alcohol intake contributes, together with other risk factors, to the development of breast cancer.^64^ Furthermore, drug use disorders reduce adherence to screening programs, leading to late detection and advanced-stage diagnosis of both breast and cervical cancer.^65,66^

**Figure 3.**
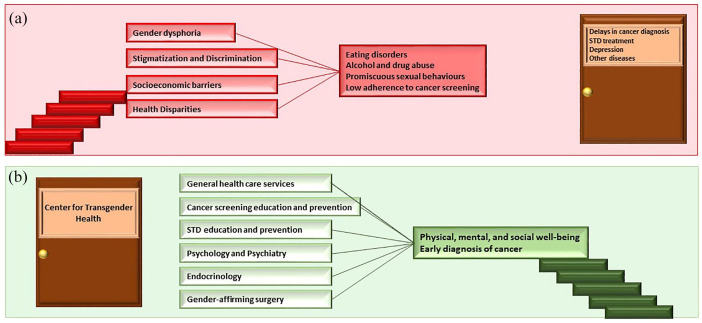
(a) In transgender people, the distress provoked by gender dysphoria, stigmatization, discrimination, socioeconomic barriers, health disparities can lead to eating disorders, alcohol and drug abuse, and promiscuous sexual behavior, which are risk factors for several diseases, included cancer. These factors, along with low adherence to cancer screening, may also contribute to delays in cancer diagnosis. (b) A center for transgender general health, including different medical specialties and preventive health services, may help transgender people overcome the distress, contributing to physical, mental, and social well-being and early diagnosis of cancer. STD, sexually transmitted disease.

Risk factors and predisposing conditions, which contribute to several diseases, including cancer, are reported in Figure 3. In the same figure, we suggest to implement the organization of transgender health centers, offering general health services and preventive health services to provide comprehensive and not only gender-affirming care.

To our knowledge, the present report describes the only case of a transgender man who developed two synchronous female cancers, which are in some way related to lifestyle, sexual behavior, and poor adhesion to screening programs. The uniqueness of our report certainly represents a major strength of our work, contributing to the published literature on health disparities and cancer risk factors in transgender individuals. Some limitations must be acknowledged, including the lack of details on the germline BRCA status, and on the personal experience with sex discrimination. Moreover, the review of the literature on this topic provides fragmentary information and no robust evidence, due to the retrospective nature and the small size of most of the cited studies.

## Conclusion

In conclusion, the knowledge about transgender specific health issues, including cancer, is still limited. Specific cancer guidelines are often confusing and the medical literature on this topic is insufficient. The impact of exogenous hormone administration on the development of cancer in transgender men remains unclear. Moreover, adherence to cancer screening procedures, including diagnostic imaging, Pap test, and HPV vaccination, is often suboptimal or unknown, due to structural and individual barriers, all of which are factors contributing to delays in cancer diagnosis and subsequent treatments.

The transgender population is increasing over time and, consequently, more personalized care pathways are urgently needed. The aim of the present report is to strongly advocate for screening prevention in the transgender population, overcoming intrapersonal and social barriers for both transgender people and healthcare professionals.

## Supplemental Material

sj-docx-1-tam-10.1177_17588359241259466 – Supplemental material for Breast and cervical cancer in transgender men: literature review and a case report Supplemental material, sj-docx-1-tam-10.1177_17588359241259466 for Breast and cervical cancer in transgender men: literature review and a case report by Francesca Sofia Di Lisa, Alice Villa, Lorena Filomeno, Teresa Arcuri, Benito Chiofalo, Giuseppe Sanguineti, Laura Pizzuti, Eriseld Krasniqi, Maddalena Barba, Domenico Sergi, Francesco Lombardo, Francesco Romanelli, Claudio Botti, Giovanni Zoccali, Gennaro Ciliberto and Patrizia Vici in Therapeutic Advances in Medical Oncology

## Declarations

**Ethics approval and consent to participate:** The manuscript was approved by Ethic Committee Lazio Area 5 (N° 66/IRE/23, Case Report Notification). Written consent for participation was obtained from the patient.

**Consent for publication:** Written informed consent for publication of clinical details and clinical images was obtained from the patient.

**Availability of data and materials:** Not applicable.
